# Bacteriophage isolation from human saliva: a pilot study with high school students

**DOI:** 10.1080/07853890.2021.1896919

**Published:** 2021-09-28

**Authors:** Teresa Nascimento, Matilde Marvão, Joana Bugalho, Marta Bastos, Andreia Luz, Paulo Maurício, Nuno Taveira

**Affiliations:** a Centro de Investigação Interdisciplinar Egas Moniz (CiiEM), Egas Moniz Cooperativa de Ensino Superior, Caparica, Portugal; b Colégio Valsassina, Lisboa, Portugal; c Research Institute for Medicines (iMed.ULisboa), Lisboa, Portugal

## Abstract

**Introduction:**

The microbiome of the human oral cavity is composed of numerous and diverse bacteria, archaea, eukarya and viruses [1]. Bacteriophages (abbreviated phages) are bacterial viruses that can attack and kill a target bacterium within minutes of infection. Very little is known about the impact of phages on the ecology of the oral microbiome and the aetiology of diseases of the oral cavity [2]. The lytic capacity of some phages suggests, that this may be promising antimicrobial agents that could be used to prevent or treat oral diseases [3]. The study aimed to isolate bacteriophages specific for *Streptococcus mutans* (causal agent of dental caries) and *Enterococcus faecalis* (causative agent of persistent apical periodontitis) from human saliva with the engagement of high school students in scientific research.

**Materials and methods:**

Saliva samples were collected from 61 healthy donors, undergraduate students from Valsassina College, Lisbon, Portugal. All samples were examined for the presence of phages using the agar overlay method. The study was approved by the Egas Moniz Ethics Committee (approval number 636) and written informed consent was obtained from all subjects.

**Results:**

Three to five days after inoculation with *E. faecalis*, uniform turbid lysis zones were generated by saliva samples collected from 6 of 61 individuals (9.8%). No plaques for *S. mutans* were evident after direct plating of the material.

**Discussion and conclusions:**

It was possible to isolate *E. faecalis*, but not *S. mutans* bacteriophages. Our data is similar in prevalence to previous studies who also attempted to isolate lytic bacteriophage from oral *E. faecalis* [4]. The presence of *E. faecalis* phages in the saliva of healthy individuals suggests that they may play a role in the control of this bacterium in the oral cavity.
